# Onset of Addison Disease appeared during the first trimester of a twin pregnancy: A case report

**DOI:** 10.1002/ccr3.3784

**Published:** 2021-05-05

**Authors:** Angela Tagetti, Denise Marcon, Paolo Moghetti, Giovanna Spiazzi, Cristiano Fava, Pietro Minuz

**Affiliations:** ^1^ Department of Medicine Section of General Medicine and Hypertension University of Verona Verona Italy; ^2^ Department of Medicine Section of Endocrinology, Diabetes and Metabolic Disease University of Verona Verona Italy

**Keywords:** Addison disease, pregnancy, therapy, twins

## Abstract

Addison Disease is an uncommon, life‐threatening condition affecting people at any age, including women during pregnancy. If left untreated, the disease can be rapidly fatal, but the prognosis is good if promptly recognized and hormones are replaced.

## Graphical abstract

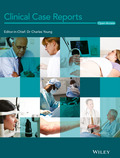

## 1 INTRODUCTION

This case describes the onset of Addison disease in a pregnant woman during the first trimester of a twin pregnancy and gives useful information about the initial and chronic treatment of Addison disease during pregnancy aimed at preserving the mother and fetuses’ health. A 32‐year‐old woman at 16 + 3 gestational weeks, with celiac disease and Hashimoto thyroiditis, was admitted to the Obstetrics division for persistent vomiting with difficulty feeding, weakness, and mild pelvic pain. At admission, the patient's blood pressure was 90/60 mmHg and body temperature 35.4°C. Serum sodium was 117 mmol/L. After the hypothesis of undertreated hypothyroidism was discarded, Addison disease was diagnosed, and intravenous therapy with hydrocortisone was initiated. After five days, hemodynamic stabilization and normalization of laboratory tests were obtained. Intravenous therapy was switched to oral hydrocortisone and fludrocortisone. Fetuses' and mother's conditions were strictly monitored, and the pregnancy ended at 35 + 2 weeks because of premature rupture of membranes. Newborns were in good health status. Fifteen months after delivery, clinical conditions have improved, and hydrocortisone has been gradually reduced to 25 µg/d. Rapid diagnosis, correct adjustment of glucocorticoid, and mineralocorticoid replacement therapy and fetus and mother's close monitoring were the critical point of the successful management. Addison Disease is an uncommon, life‐threatening condition that can affect people at any age, including women during pregnancy. If left untreated, the disease can be rapidly fatal, but the prognosis is good if promptly recognized and hormones are replaced.

Primary adrenal insufficiency (PAI) is a rare but potentially severe condition.^1^ PAI is much more challenging to diagnose and severe if it occurs during gestation. Current guidelines dictate PAI management during pregnancy, but no indications are offered for new‐onset PAI.^1^, ^2^ Since untreated PAI in pregnant women is associated with high mortality, whereas for adequately treated patients, normal pregnancy course and the outcome are expected, early recognition and diagnosis are crucial.^1^

### 1.1 Case presentation

A 32‐year‐old Caucasian woman at 16 + 3 weeks’ gestation of a dichorionic, diamniotic twin pregnancy, without a history of smoking or drug abuse, was admitted to the Obstetrics division for persistent severe vomiting with difficult feeding, weakness, and mild pelvic pain. The patient had no significant family history. In 2014, the woman was diagnosed with Celiac disease and lactose intolerance treated by gluten and lactose‐free diet. In 2012, she was diagnosed with Hashimoto's thyroiditis and treated with administration of levothyroxine 475 µg/wk. For failure of embryo attachment at 7 + 4 weeks of gestation, she was treated with 200 mg daily of intravaginal progesterone. Treatment was then shifted to 341mg of intramuscular 17‐α hydroxyprogesterone caproate (Lentogest®) every 3 days for topical intolerance from week 9. She appeared healthy at admission, and physical examination was normal except for low blood pressure (90/60 mmHg) and low body temperature (35.4°C). The heart rate was 100 bpm/min.

### 1.2 Diagnostic assessment

Laboratory tests at admission showed serum sodium 117 mmol/L, potassium 4.68 mmol/L, chloride 64 mmol/L. TSH and thyroid hormones showed a well titrated therapy (TSH 1.19 mUI/L (normal range: 0.35‐3 mUI/L), FT3 4 pg/mL (2.1‐4.9 pg/mL), FT4 11.5 ng/L (8‐19 ng/L). Vomiting ceased a few hours after the admission, and hyperemesis gravidarum was excluded. Further hormonal tests showed increased plasma‐ACTH 648 pg/mL (normal values 8.5‐50), plasma‐cortisol 14.5 μg/dL (4‐25), plasma renin >500 μU/mL (3.3‐41), serum aldosterone 65 pg/mL (35‐300) in orthostatic and 67 pg/mL (7.5‐150) in supine position. Other laboratory tests were normal, except for serum sodium that was persistently low and gradually increased with saline infusion and normalized within six days.

### 1.3 Treatment

After the diagnostic hypothesis of adrenal insufficiency arose, the patient was treated with 100 mg of hydrocortisone intravenously three times a day, then 50 mg tid iv because of hyperglycemia. After five days, oral therapy was started with hydrocortisone 10 mg tid plus fludrocortisone 100 µg, up‐titrated to 200 µg in 6 days.

### 1.4 Outcome and follow‐up

The pregnancy proceeded without complications. The patient was clinically stable, with normal plasma electrolytes, glucose, and clinostatic and orthostatic BP. She was admitted to the Hospital for vomiting and diarrhea during the 29th week of pregnancy and was discharged home with the diagnosis of viral gastroenteritis. On that occasion, high dose hydrocortisone was administered (100 mg every 6 hours intravenously) for the first 24 hours; then, the usual oral dosage regiment for corticosteroids was restored and maintained until delivery. Fetal ultrasound examination was normal. Caesarean section for premature rupture of membranes was performed at 35 + 2 weeks without complications, after the iv administration of 12mg of betamethasone for the prevention of respiratory distress syndrome in the new‐born. Two females, 2000 g and 2370 g of weight, presenting normal umbilical cord, plasma pH and Apgar score 10/10, were born. During labor, the mother was treated with iv hydrocortisone (100 mg bolus), followed by infusion of 200 mg for 24 hours along with fluids administration. Thirty‐six hours after delivery, oral therapy was restored at twice the dose taken during pregnancy (hydrocortisone 20 + 20 + 20 mg, fludrocortisone 400 µg). After another day, the dose was reduced to that used in the last weeks of gestation. Five days after delivery, the dosage of fludrocortisone was tapered at 150 µg. One week after the Caesarean section, the patient was discharged home with the following substitutive therapy: hydrocortisone 10 + 5+5 mg + fludrocortisone 150 µg. During the next 5 months, fludrocortisone was gradually reduced to 50 µg/d for edema and low plasma renin. Measurement of serum and urinary cortisol showed that celiac disease did not affect hydrocortisone intestinal absorption. After 16 months, serum sodium, potassium, and glucose levels were normal, urinary cortisol was within the normality (70 nmol/die), and serum‐cortisol peak level was 636 nmol/L, and therapy was therefore confirmed. The patient reported no difficulties in assuming treatment, and no adverse effects were notable.

## 2 DISCUSSION

A crucial point in this patient's management was the rapidity in recognizing and treating the adrenal insufficiency. The differential diagnosis included at least three other disorders that share the same symptoms and signs: uncontrolled hypothyroidism, hyperemesis gravidarum, and a side effect of progesterone. The first was rapidly ruled‐out based on the patient's clinical features and the normality of thyroid hormones and TSH; additionally, hyponatremia is sometimes associated with hypothyroidism, but this may be mainly in patients with severe primary hypothyroidism and myxedema.^3^, ^4^, ^5^ Hyperemesis gravidarum was also excluded because vomiting ceased with fluid repletion. Progesterone use can be potentially associated with hyponatremia because it may diminish the effects of aldosterone in the renal tubule and decrease sodium reabsorption with the following increase in mineralocorticoid secretion from the adrenal cortex.^6^ It is essential to underline that hydrocortisone's dose was slightly modified, while mineralocorticoid dosage had undergone a more critical adjustment. This fact may be due to the anti‐mineralocorticoid action of progesterone, which implies an adaptive response. During pregnancy, there is an activation of renin‐angiotensin‐aldosterone and an increase in progesterone concentration, which competes with aldosterone for binding to the type 1 corticosteroid receptor, resulting in reduced responsiveness to the sodium‐retaining effect of aldosterone.^7^ In healthy women, increased plasma renin activity and aldosterone levels compensate for progesterone action. But in Addisonian pregnant patients, a larger dosage of mineralocorticoid replacement may be indicated.^7^

The coexistence of Addison, Hashimoto, and celiac diseases is consistent with the diagnosis of type II polyglandular autoimmune syndrome, an autoimmune disorder of polygenic inheritance, and pleomorphic phenotype.^8^ The clinical picture was also challenging because of the twin pregnancy, lack of recommendation for appropriate clinical management and the initial replacement therapy during pregnancy. Current guidelines ^1^ address the dose adjustment during pregnancy, but initial replacement therapy in new‐onset PAI during pregnancy is not specified. Current guidelines suggest close monitoring for clinical symptoms and signs of glucocorticoid excess or under‐replacement therapy at least once per trimester for pregnant patients with known PAI. During the third trimester, dosage of hydrocortisone should be augmented. Hydrocortisone should be used over cortisone acetate, prednisolone, or prednisone because it is not inactivated in the placenta. During the active phase of labor, a similar dosage to that used in major surgery is recommended. A case report suggests rapid glucocorticoid replacement with iv 100‐200 mg of hydrocortisone as a single bolus and 50‐100 mg boluses every 6‐8 hours during the acute period.^9^ In this case, the dose of replacement therapy is modulated, primarily based on serum potassium, serum sodium levels and clinical response as assessed by measuring blood pressure and heart rate. Our clinical case highlights the importance of close collaborations between different medical specialists (internal medicine, endocrinology, obstetrics). It is crucial in detecting and treating such a complex and life‐threatening condition.

## CONFLICT OF INTEREST

No conflict of interest to declare.

## AUTHOR CONTRIBUTION

Angela Tagetti and Denise Marcon collected clinical and laboratory data and drafted the manuscript; Cristiano Fava and Pietro Minuz were involved in drafting the manuscript, in the diagnosis and in the initial treatment of the disease; Paolo Moghetti and Giovanna Spiazzi were the patient's physicians. All the authors contributed to the critical review of the paper.

## ETHICAL APPROVAL

Ethical committee Of Verona and Rovigo approved the study in April 27, 2020 (Project number 2666CE; Protocol number n. 23 431).

## Data Availability

None.
